# Fully Printed Flexible Polystyrene/Graphite-Based Temperature Sensor with Excellent Properties for Potential Smart Applications

**DOI:** 10.1021/acsomega.4c09548

**Published:** 2025-01-23

**Authors:** Ahmad Al Shboul, Mohsen Ketabi, Jenner H. L. Ngai, Daniella Skaf, Simon Rondeau-Gagné, Ricardo Izquierdo

**Affiliations:** † Department of Electrical Engineering, École de Technologie Supérieure (ETS), 1100 Notre-Dame St W, Montreal, Quebec H3C 1K3, Canada; ‡ Security and Disruptive Technologies (SDT) Research Centre, National Research Council of Canada, 1200 Montreal Road, Ottawa, Ontario K1A 0R6, Canada; § Department of Chemistry and Biochemistry, Advanced Materials Centre of Research, University of Windsor, Windsor, Ontario N9B 3P4, Canada

## Abstract

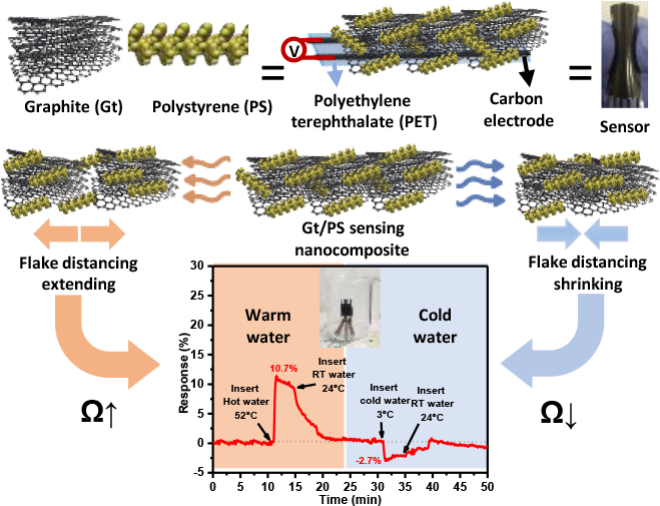

This study presents an innovative temperature sensor based on a thermistor nanocomposite of graphite (Gt) and polystyrene (PS). The sensor exhibited notable thermal stability and film integrity, offering two distinct linear response regions within the tested temperature range of −10 to 60 °C. It demonstrated a sensitivity of 0.125% °C^–1^ between −10 and 10 °C, followed by another linear response with a sensitivity of 0.41% °C^–1^ from 20 to 60 °C. Furthermore, it exhibited a response/recovery time of 0.97/1.3 min at a heating/cooling rate of 60 °C min^–1^. The sensor maintained minimal baseline drift even when subjected to varying humidity levels. We assessed its mechanical flexibility and stability for hundreds of bending cycles at a bending angle of 30°, adapting to dynamic environmental conditions. The sensor’s thermomechanical test (response to mechanical stress under temperature fluctuations) underscored its adaptability over a temperature range of −10 to 60 °C. Notably, it displayed excellent chemical stability, maintaining consistent performance when subjected to harsh environmental conditions like exposure to corrosive gases and prolonged immersion in tap water. Real-world tests demonstrated its practical utility, including precise temperature measurements in solid objects and breath temperature monitoring. These findings suggest promising applications in healthcare, environmental monitoring, and various IoT applications.

## Introduction

The growing focus on flexible and wearable designs is a significant advancement in temperature sensor research. This shift is evident in numerous reviews exploring flexible sensors,^1,2^ graphene-based wearable sensors,^3^ and textile-integrated technologies.^4^ The emphasis on flexibility and wearability represents a move toward more user-friendly and adaptable temperature sensing solutions, particularly for health monitoring and IoT applications.^5^

Flexible temperature sensors have revolutionized temperature monitoring by offering several advantages over traditional rigid sensors,^6^ making them indispensable in scenarios where rigid designs are impractical.^6,7^ These sensors are thin, lightweight, and efficient, capable of withstanding physical deformation without compromising sensitivity, accuracy, repeatability, or stability.^8^ Moreover, their ability to conform seamlessly to surfaces enhances comfort and usability.^9^

Flexible temperature sensors are the subject of extensive research efforts for body temperature monitoring in wearable and biomedical applications, such as smart electronic skin (E-skin),^10−15^ and smart electronic bandage (E-bandage).^16−19^ These inventions provide noninvasive temperature monitoring for patients who require continuous monitoring to screen vital signs without discomfort. This feature is handy for patients in critical care units or undergoing surgery who require continuous temperature monitoring. Accordingly, flexible and wearable temperature sensor development has become a research hotspot for scientific researchers. The most recent BCC Research report indicates that, at a compound yearly growth rate (CAGR) of 4.3% from 2023 to 2028, the demand for temperature sensors in the global markets would increase from $7.3 billion in 2023 to $9.0 billion by the end of 2028.

Flexible temperature sensors embedded diverse sensing materials, such as polymers,^7,20^ graphene,^21−24^ carbon nanotubes (CNTs),^25,26^ metal oxide semiconductors (MOs),^27^ and conductive inks.^28^ The choice of materials depends on the specific application and the desired properties of the sensor, such as sensitivity, response time, and temperature resolution. Graphene-based temperature sensors have advantages such as high thermal conductivity and large specific surface area,^3^ while metal oxide-based temperature sensors have good electrical performance and are suitable for low-temperature applications.^27^

However, the structure, material, cost, fabrication process, and performance are essential factors of flexible temperature sensors, and previous studies have found that they significantly influence the performance of sensors. Graphene and CNT-based temperature sensors face low sensitivity, poor stability, and high noise levels.^21^ The difficulty of controlling the alignment of CNTs affects the sensitivity and stability of CNT-based temperature sensors.^29^ Polymer and MOs-based temperature sensor sensitivity can be affected by environmental factors such as relative humidity (RH), gas poisoning, and aging.^30−32^ Additionally, the thickness and quality of the sensing layer can affect the sensor’s sensitivity.^27,33^ Moreover, the fabrication of flexible temperature sensors on a large scale is still a challenge due to the relatively high cost of production.^21,30,34^ Ongoing research and development in flexible temperature sensors continue to address these challenges by using hybrid materials, optimizing sensor structures, and developing new fabrication techniques.^23,27,35−37^

Polystyrene (PS) has recently garnered significant attention in temperature sensing, particularly optical-fiber temperature sensors.^38−41^ This interest arises from its favorable coefficient of thermal expansion and thermo-optic coefficient.^38^ PS is a synthetic thermoplastic polymer that can be melted and reformed repeatedly without undergoing chemical changes.^42^ Accordingly, several notable studies have explored the potential of PS-based sensors. Chen et al. developed a fiber-optic temperature sensor utilizing a PS microsphere at the fiber tip. Their sensor exhibited a sensitivity of −0.61796 nm °C^–1^ within a temperature range of 20–70 °C.^41^ Salunkhe et al. reported a PS-coated optical fiber temperature sensor, achieving a high sensitivity of 439.89 pm °C^–1^ from 25 to 100 °C.^38^ Neitzert et al. ventured into fabricating an optical temperature sensor employing a PS/multiwalled CNTs (MWCTs) nanocomposite, demonstrating a negative temperature coefficient of resistance (TCR) from room temperature (RT) up to about 50 °C.^40^ Malekie et al., on the contrary, developed a calorimetric temperature sensor using the PS/MWCTs nanocomposite, showcasing a positive TCR in a temperature range of 20–50 °C.^41^

While these studies have collectively highlighted the versatility and potential of PS-based materials in developing temperature sensors with varying TCR behaviors, It is important to note that their applications have mostly been restricted to temperatures above RT. This limitation poses challenges when considering the sensor’s applicability in scenarios requiring precise measurements at lower temperature ranges. Additionally, despite their performance, there is a lack of comprehensive data regarding the cost of sensor fabrication and the feasibility of large-scale production. Addressing these aspects will be crucial in determining the practicality and accessibility of PS-based temperature sensors for a broader range of applications.

This paper presents a straightforward approach to addressing persistent issues in flexible temperature sensors. We were able to create a flexible temperature sensor with improved properties by utilizing a sustainable-based nanocomposite made of PS and graphite (Gt) flakes powder (flake size <20 μm). By adopting these sustainable materials, we advance toward a greener future and foster innovation for a more sustainable and resilient world. We used the doctor blade coating, a large-scale printing technique, to create our printed and flexible sensors. This careful integration of materials and fabrication processes ensures optimal performance and longevity, putting our devices at the forefront of sensor technology. By seamlessly combining these carefully selected materials with advanced techniques, we unlock new possibilities and revolutionize the field of noninvasive and accurate measurements. Our innovative approach enhances the functionality of our sensor and opens doors to previously unexplored possibilities, pushing the boundaries of what is achievable.

## Experimental Section

### Materials and Methods for Ink Formulation

**Figure 1:**
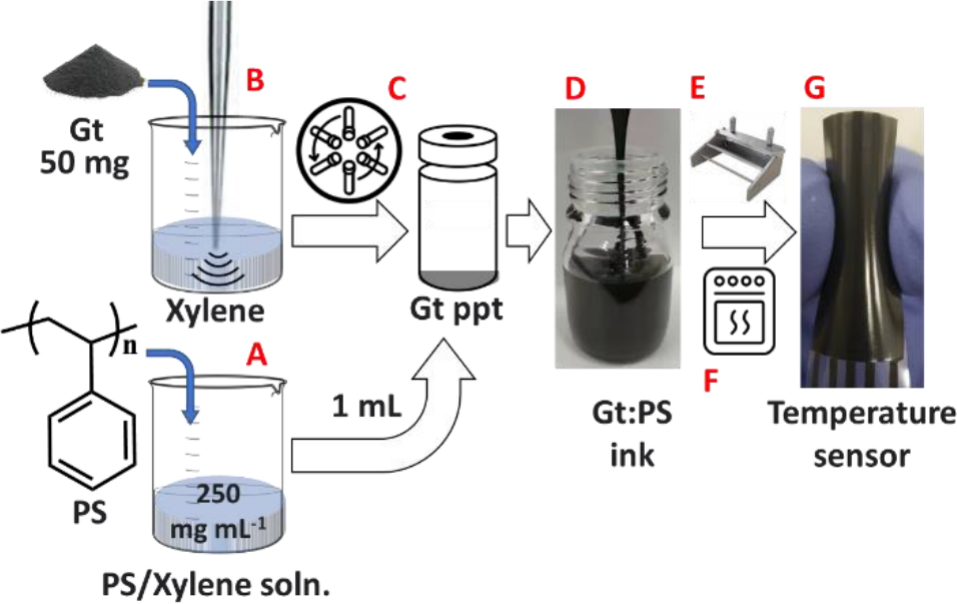
Schematic illustration of the process for fabricating the wearable temperature sensor. (A) Preparation of PS solution in xylene solvent. (B) Ultrasonication of Gt powder in xylene for 10 min. (C) Add 1 mL of PS solution (250 mg mL^–1^) to the Gt precipitate after centrifugation at 1000 rpm for 10 min. (D) Photograph of the Gt/PS nanocomposite ink after mixing on a magnetic stirrer for 1 h. (E) Prepare a thin Gt/PS nanocomposite film using the doctor blade method on prescreen printed carbon electrodes on a PET substrate. (F) Curing the thin film at 90 and 180 °C for 15 min each. (G) Photograph of the fabricated temperature sensor.

The temperature sensor preparation procedure (Figure 1) was modified from our preceding reports.^43,44^ First, a PS (Mw ∼ 192,000 Da, Sigma-Aldrich) solution with a concentration of 250 mg mL^–1^ (viscosity of 650 mPa·s and surface tension 28.3 Mn m^–1^ at 24 °C) was prepared in xylene (ACS reagent, 98.5%, Sigma-Aldrich) (Figure 1A). Simultaneously, a 500 W sonication probe was used to disperse 50 mg of Gt flakes powder (<20 μm flake size, Sigma-Aldrich) in xylene for 10 min (Figure 1B). The sonication process was carried out in an ice bath to prevent overheating, and a magnetic stir bar was used to ensure homogeneous Gt flakes dispersion. Following the completion of the sonication, the Gt dispersion was precipitated at the vial’s bottom by centrifugation at 1000 rpm for 1 min (Figure 1C). Then, ink was made by adding 1 mL of the PS solution (250 mg mL^–1^) to the Gt flakes precipitate and stirring vigorously for 1 h at RT with a magnetic stir bar (Figure 1D). The Gt flakes concentration in the ink was estimated at 50 mg mL^–1^.

### Temperature Sensor Fabrication

The temperature sensor was produced by applying the formulated nanocomposite ink directly onto screen-printed carbon electrodes, which were deposited into a polyethylene terephthalate (PET) substrate using the doctor blade technique, as shown in Figure 1E. The PET substrate serves as a flexible base for the sensing layers, offering mechanical flexibility for applications like wearable devices. It provides moderate thermal stability, withstanding the 180 °C curing process (as will be discussed next) without deformation, ensuring structural integrity. Additionally, PET acts as an electrical insulator, preventing unintended current flow and maintaining sensor accuracy.

The sensor was cured in an oven in two stages at two different temperatures. The sensor was first cured at 90 °C for 15 min to evaporate the xylene solvent. The sensor was then cured for 15 min at an optimized temperature of 180 °C (Figure 1F). Details can now be found in the Supporting Information about systematically optimizing the curing temperature by studying the decomposition behavior of pure PS films at various curing temperatures using Thermogravimetric analysis (TGA, Figure S1). Data on the sensor morphology at different curing temperatures, assessed through scanning electron microscope (SEM, Figure S2) analysis, are also included in the Supporting Information. Raman spectra (Figure S3) were conducted to validate the Gt’s graphitization degree (i.e., sp^2^-structure quality) as measured by the intensity ratio of G and D bands. These details validate the fabrication process but are not central to the study’s main conclusion.

Figure 1G depicts the finished temperature sensor. The screen-printed carbon electrodes were 20 mm long, 0.5 mm wide, and 5 μm thick, with a 1 mm spacing between the carbon bars. The sensing thin film measured 15 mm in length, 3 mm in width, and 5 μm in thickness. Once the sensitive film was deposited, the resistance between two carbon electrodes in ambient air was between 150 and 200 kΩ as measured by a Keithley sourcemeter (Keithley 2601A Sourcemeter).

### Characterization

The surface morphology and composition of the fabricated sensor were characterized by the SEM (Hitachi SU-8230, Japan) with energy-dispersive X-ray spectroscopy (SEM-EDS) (Bruker, QUANTAX FlatQUAD, Germany). TGA (TA Instruments, TGA Q500, USA) was conducted under air using a heating rate of 10 °C min^–1^, ranging from 30 to 1000 °C. This analysis enabled the measurement of the change in the sample’s weight as a function of temperature and provided insight into the thermal stability and composition of the material being analyzed. Raman spectra, spanning the range of 100–3200 cm^–1^ at RT in the air, were obtained using a Renishaw Raman microscope (inVia) equipped with a 532 nm incident laser. The viscosity was measured using a viscometer (A&D, SV-10, Japan), and the surface tension was measured using a dynamic tensiometer (Dataphysics, DCAT11, Germany). Fourier transform infrared spectroscopy (FTIR, Thermo Scientific 4700) was employed to investigate the xylene’s drying rate by depositing a drop of the solvent onto a diamond crystal used as a sensor, measuring the solvent’s absorbance versus time.

### Evaluation of the Sensing Performance

The performance of a resistive temperature sensor made from Gt/PS nanocomposites was evaluated in real-time through a series of experiments designed to test its efficiency under various conditions. The comprehensive sensor evaluation included sensitivity, response and recovery times, dynamic temperature response, antihumidity characteristics, chemical and physical stability, mechanical flexibility, thermomechanical properties, and compatibility testing in real-world applications. These extensive tests provided invaluable insights into the sensor’s performance, highlighting potential areas for enhancement. It is crucial to validate the efficacy of this flexible temperature sensor and optimize its performance across various applications. Therefore, we strongly recommend treating these tests as essential performance metrics, ensuring they are rigorously conducted and thoroughly documented in future research.

A Nextron probe station (Peltier type sample stage, Korea) was used to assess the sensor’s performance within three distinct temperature ranges: −10 to 100, −10 to 80, and −10 to 60 °C, all maintained at a 10% RH (dry) level. Furthermore, the temperature sensor was subjected to rigorous testing, including multiple heating and cooling cycles from −10 to 60 °C, conducted under 10% RH (dry) conditions. Testing the sensor at low temperatures of −10 °C ensures the sensor’s suitability for applications in extreme cold conditions, such as refrigeration, cold storage, or outdoor environments in cold climates like Canada, where winter temperatures typically range from −10 to −20 °C and can occasionally drop as low as −40 °C. This lower temperature enables assessment of the Gt/PS nanocomposite’s structural and electrical properties, confirming functionality without degradation or resistance drift and showcasing the sensor’s robustness and versatility.

Changes in electrical resistance as a function of time were measured using a programmable multimeter connected to a PC via an Arduino card. The sensor’s response to temperature variations was evaluated through a series of consecutive temperature steps within the range of −10 to 60 °C, conducted using the Nextron probe station at RH ≈ 10%. Initially, the station was configured to establish reference conditions at 20 °C and RH ≈ 10%. Starting from this point, the target temperatures were reached by gradually increasing or decreasing the temperature at a rate of 20 °C per minute (20 °C min^–1^). Once the desired temperature was reached was maintained for 5 min before proceeding to the next temperature increment. After reaching the highest (60 °C) or lowest (−10 °C) target temperatures, the sensor was subjected to a controlled cooling or heating process through a series of sequential temperature steps. This process continued until the full cycle was completed, ultimately returning to a reference temperature of 20 °C. This process was repeated under RH ≈ 80%, simulating wet conditions, and the sensor’s performance was subsequently compared between dry and wet environments. Validating the sensors’ response below −10 °C posed considerable challenges during the experiments, primarily due to the limitations inherent in the Nextron Probe Station’s setup.

Eq 1 was used to calculate the sensor response, where *R*_0_ and *R*_*T*_ represent the electrical resistances for the sensor at 20 °C (*T*_0_) and the target temperature (*T*), respectively. Eq 2 was used to calculate the temperature sensor’s sensitivity, as determined by its TCR.
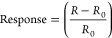
1
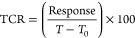
2

The dynamic temperature responses of the fabricated sensor were analyzed to assess its sensitivity, accuracy, and response time to rapid temperature fluctuations. The sensor was subjected to heating/cooling temperature steps, starting from 20 °C and RH ≈ 10%, with a rate of 20 °C min^–1^ until reaching the targeted temperature (ex. 30 °C). The temperature was maintained at the temperature for 5 min before recovering to 20 °C again. This process was done for temperatures −10, 0, 10, 20, 30, 40, 50, and 60 °C.

The sensor’s response and recovery times were assessed by measuring the duration required to transition between −10 and 60 °C, with recovery time indicating the period needed to return to −10 °C. These key time points were determined when the sensor reached 90% of the total resistance change. To comprehensively evaluate the sensor’s performance, tests were conducted for various heating and cooling rates of 20, 40, and 60 °C min^–1^, as the Nextron probe station’s heating and cooling rates significantly impact the sensor’s response and recovery times.

The sensor’s accuracy in detecting subtle temperature changes was evaluated through experiments involving ±3 °C temperature variations at 0, 20, 40, and 60 °C, with relative humidity maintained at approximately 10%. This thorough examination enabled us to measure and analyze the sensor’s response to subtle temperature changes, offering valuable insights into its accuracy and precision in temperature measurement.

### Chemical and Physical Stabilities

We conducted two separate investigations to thoroughly evaluate the sensor’s chemical and physical stabilities. First, we assessed its resistance to electrical changes after exposure to 4 ppm of corrosive gases at ambient conditions, including hydrogen (H_2_), nitrogen dioxide (NO_2_), sulfur dioxide (SO_2_), ammonia (NH_3_), and hydrogen sulfide (H_2_S). These experiments were conducted to ascertain the impact of these gases on the sensor’s temperature measurements and to identify and rectify any potential errors stemming from gas interference.

In a parallel approach, a batch of identical sensors was immersed in tap water at RT for several weeks, simulating lousy storage conditions. Exposure to this condition can cause hydrolysis, oxidation, expansion, or deformation of sensing materials, resulting in material distortion or destruction that affects its sensing performance. Individual sensors were extracted from the group weekly, dried in the oven at 40 °C for 10 min before the test to evaporate water residues, meticulously inspected under SEM, and subjected to temperature-sensing assessments. This examination aimed to determine the extent to which the sensors’ structural integrity was maintained compared to freshly prepared temperature sensors. Furthermore, it pursued to determine the sensor’s ability to replicate its standard temperature-sensing performance throughout the temperature ramping process, which spanned from −10 to 60 °C.

### Thermomechanical Properties Characterization

Our prior work^40,41^ thoroughly analyzed the sensors’ mechanical flexibility and thermomechanical properties. To assess the critical angle at which the sensor might experience electrical failure, we subjected it to gradual bending, with increments of 5°, while monitoring the change in electrical resistance, calculated using eq 1. Subsequently, we evaluated the sensor’s mechanical flexibility through repetitive bending cycles, pushing it to its critical angles at a bending rate of 6° per second (6° s^–1^). Throughout these assessments, we maintained a bias voltage of 6 V, utilizing a Keithley sourcemeter (Keithley 2601A Sourcemeter), and presented the results as changes in electrical resistance, as calculated by eq 1. These experiments were conducted under ambient conditions, at RT, and with a moderate RH ≈ 30–40%.

Furthermore, dynamic mechanical analysis studies were conducted using a dynamic mechanical analyzer (DMA), specifically the TA Instruments DMA850, equipped with a liquid nitrogen purge cooler. The sensor was firmly secured during these experiments using a two-cantilever film clamp. The sample was initially cooled to −40 °C and allowed to equilibrate for 10 min. Subsequently, temperature ramp studies were initiated at 1 Hz, progressing at 2 °C min^–1^ rate until the desired target temperature of 60 °C was reached.

The flexibility assessment is paramount, as it determines the sensor’s ability to withstand stress and strain. Additionally, evaluating thermomechanical stability is crucial to gauge the sensor’s resilience against temperature fluctuations, potentially affecting the integrity of sensing layers and their adhesion to substrates.

### Sensor Evaluation for Monitoring Temperature in Breath and Solid Structures

The sensor’s practicality and reliability were thoroughly evaluated through simulations of real-life scenarios. For example, we attached the sensor to a beaker to monitor the temperature of a solid structure while simultaneously assessing its response to hot, RT, and cold water. The sensor was seamlessly integrated into an inhaler spacer tube and a Temp/RH reference sensor (DollaTek SHTC3), enabling us to monitor its response during normal and rapid breathing. We then meticulously compared the sensor’s readings with the reference sensor’s readings to thoroughly assess its performance in practical, real-world applications.

## Results and Discussion

The preparation procedure involved 10 min probe sonication of Gt flakes in xylene, resulting in the easy precipitation of Gt flakes without centrifugation. This can be attributed to the mismatch in surface energy and Hansen’s solubility parameters (HSPs) between Gt and xylene. While natural Gt has a surface energy of 62 mJ m^–2^, and HSPs of 18, 9.3, and 7.7 MP^1/2^ for dispersion (δ_D_), polar (δ_P_), and hydrogen bonding (δ_H_) solubility parameters, respectively.^45^ Xylene has a surface tension of 29.6 mJ m^–2^ and HSPs of 17.8, 1.0, and 3.1 MP^1/2^ for δ_D_, δ_P_, and δ_H_, respectively.^46^ The simple mix of the Gt precipitate with the PS solution (250 mg mL^–1^) resulted in an easy coating ink with a viscosity of 650 mPa·s and surface tension of 28.3 mN m^–1^ at 24 °C. Next, the ink was applied to the screen-printed carbon electrodes on the PET substrate using the doctor blade technique, a commonly employed method for depositing thin films. The solvent (xylene)’s rapid evaporation in the preparation process was quantified using FTIR, revealing a rapid evaporation period of approximately 2.7 min at ambient RT (Figure S4). This quick evaporation facilitated the efficient and rapid Gt/PS nanocomposite fixation onto the carbon electrodes at low temperatures.

**Figure 2:**
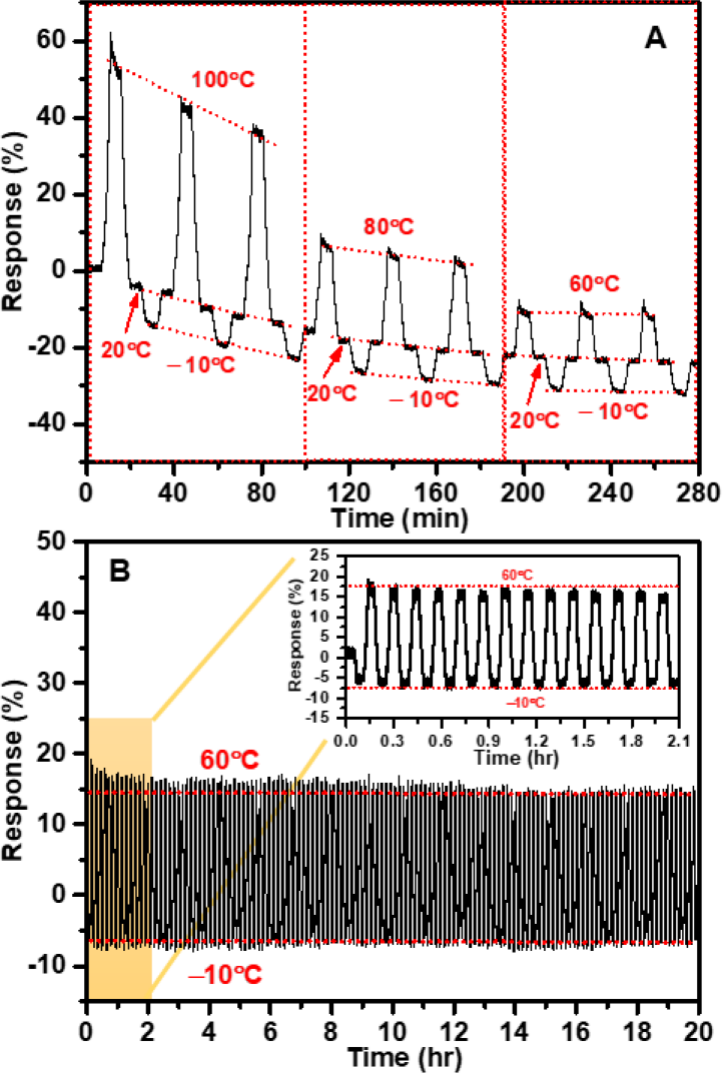
Temperature-dependent resistance changes of the Gt/PS nanocomposite-based temperature sensor: (A) Three cycles covering temperature ranges from −10 to 100, −10 to 80, and −10 to 60 °C. (B) Repeated heating–cooling cycles within the temperature range of −10 to 60 °C, and the inset zooms on the sensor’s performance during the repeated heating–cooling cycles. These tests were conducted under RH ≈ 10%.

The temperature-dependent resistance changes of the developed sensor were measured at various temperatures to assess its temperature-sensing performance. In our previous reports,^43,44^ we observed a noticeable drift during the heating and cooling cycles, which we attributed to the interference of the PET substrate in the reversible restoration of the Gt/PS nanostructure. It is worth noting that the PET substrate’s glass transition temperature (*T*_g_) can vary between 67 and 85 °C, depending on the specific grade examined.^47,48^ This observation led us to hypothesize that the softening of the PET substrate at temperatures exceeding approximately 60 °C could potentially induce distortion or twisting in the Gt/PS nanostructure, thereby causing a drift in the baseline of the sensor’s response. To confirm this hypothesis, we conducted additional tests by retesting the sensor in a Nextron test chamber for three cycles, covering temperature ranges between −10 and 100, −10 and 80, and −10 and 60 °C, all at RH of 10%. Upon analyzing the sensor’s response to temperature, we observed a distinct baseline drift within the temperature range of −10 to 100 and −10 to 80 °C. Conversely, the temperature range between −10 and 60 °C demonstrated excellent repeatability, as illustrated in Figure 2A.

To extend the operational temperature range, high-temperature plastic films such as Polyimide (PI), which offer superior thermal stability (with *T*_g_ often exceeding 300 °C) and allow operation at higher temperatures while maintaining structural integrity, could be considered in the future work plan; however, PET was deliberately chosen in this study for its cost-effectiveness, and wide availability for the specified temperature range.

When the sensor was exposed to hundreds of repeated heating and cooling cycles within the temperature range of −10 to 60 °C at a constant RH of 10% (Figure 2B), it demonstrated a notable and highly desirable feature: high reversible performance (Figure 2B (inset)). This endurance under prolonged testing conditions further highlights the sensor’s reliability and ability to maintain consistent and accurate temperature measurements within the specified operational range. This capability is crucial for various practical applications that require frequent temperature monitoring and control, including industrial processes, food processing, and medical equipment. Additionally, the sensor’s reusability could help reduce costs and waste associated with disposable sensors. Overall, its strong performance in cycled heating and cooling processes underscores its potential for practical and cost-effective applications.

**Figure 3:**
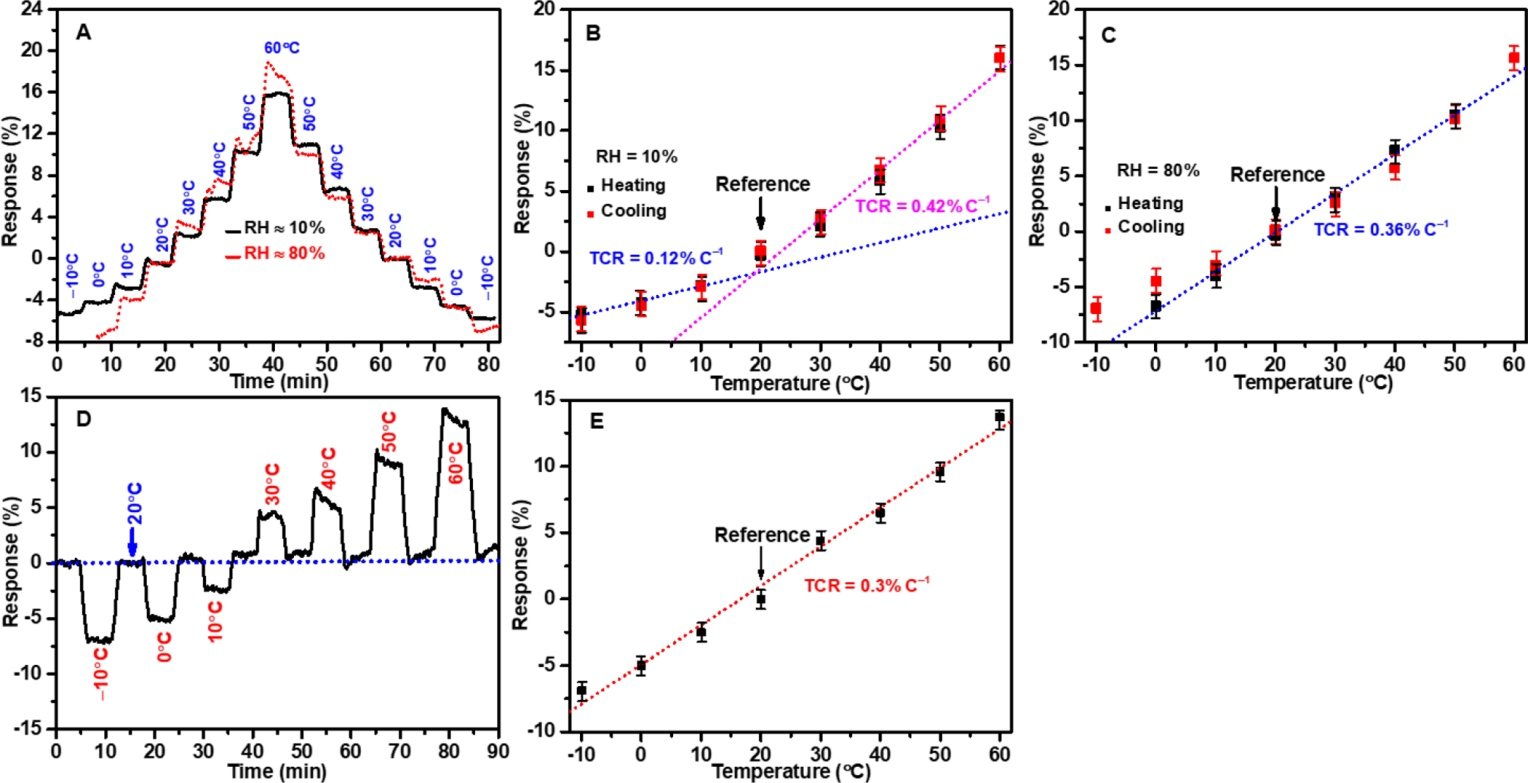
Temperature-dependent resistance changes of the Gt/PS nanocomposite-based temperature sensor: (A) step-like response to temperature from −10 to 60 °C at RH 10 and 80%, and sensor response analysis at (B) RH ≈ 10% and (C) RH ≈ 80%. (D) Dynamic response curve of the temperature sensor in uncontrolled RH conditions (RH 40–90%), and (E) analysis of dynamic response curves for the temperature sensor. Experiments used three independently fabricated sensors, each tested in triplicate, yielding nine measurements per data point. Reported values represent the average of these measurements.

The experimental results presented in Figure 3A illustrate the temperature sensor’s response to a step-like temperature change ranging from −10 to 60 °C in both dry (RH ≈ 10%) and wet (RH ≈ 80%) environments. In practical applications, varying moisture levels can impact temperature readings, so assessing the sensor in different humid levels is crucial. This evaluation can help identify any potential drift or hysteresis effects due to moisture, leading to inaccurate temperature readings.

**Figure 4:**
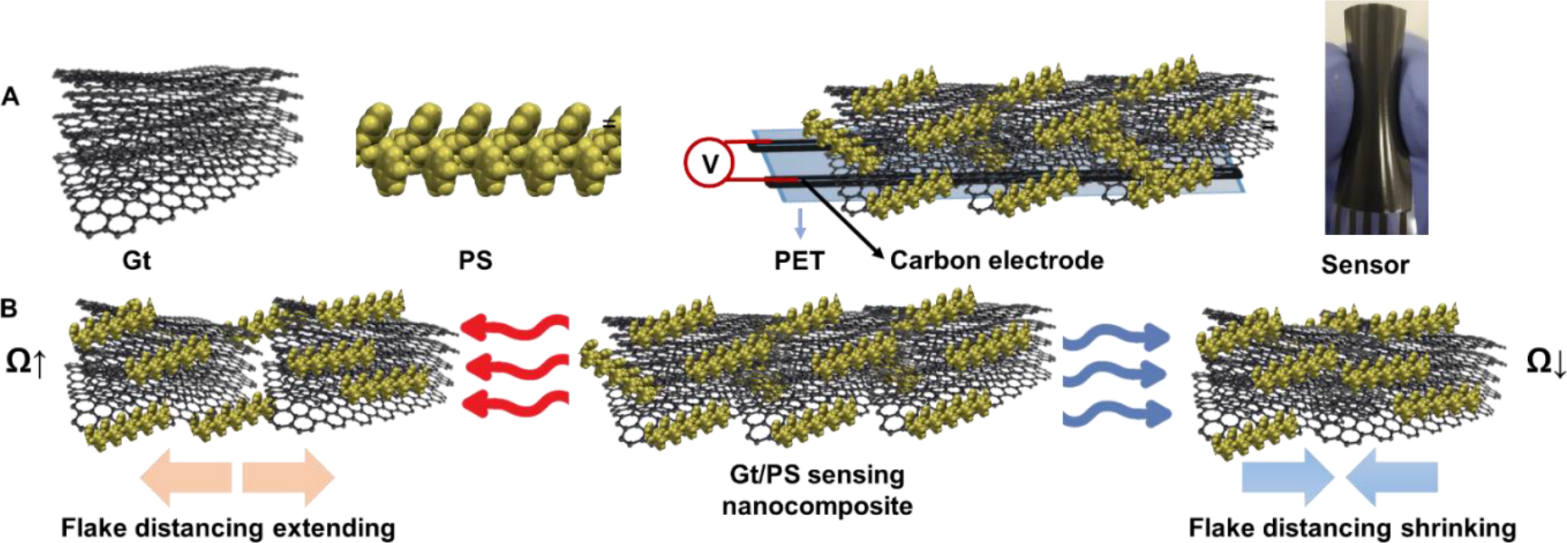
(A) Schematic representation of the Gt and PS nanocomposite coating applied over prescreen printed carbon electrodes on a PET substrate. (B) Schematic representation of the sensor’s mechanism, showing how it responds to both increasing and decreasing temperatures.

The sensor exhibited a step-like response to temperature changes (both increasing and decreasing) at both humidity environments (RH ≈ 10% and 80%). At RH ≈ 10%, the sensor showed a reversible response behavior for heating–cooling processes without significant baseline drift at different temperatures. Figure 3B shows that the sensor offers two distinct linear response regions within the tested temperature range: from −10 to 10 °C and from 20 to 60 °C. Noteworthy, the temperature of 20 °C was used as the reference point before adjusting the temperature within the range of −10 to 60 °C. The sensor recorded an initial response of −5% at −10 °C, gradually increasing to 15.9% as the temperature reached 60 °C. It demonstrated a sensitivity of TCR ≈ +0.12% °C^–1^ for the first linear response between −10 and 10 °C, followed by a higher sensitivity of TCR ≈ + 0.42% °C^–1^ for the second linear response from 20 to 60 °C. The positive TCR of thermistor material signifies that the sensor’s electrical resistance proportionally increases with rising temperatures and vice versa.^49^ The high second TCR in the sensor’s response indicates that the sensor’s output changes more rapidly with temperature in the second linear region (20–60 °C) compared to the first linear region (−10 to 10 °C).

**Figure 5:**
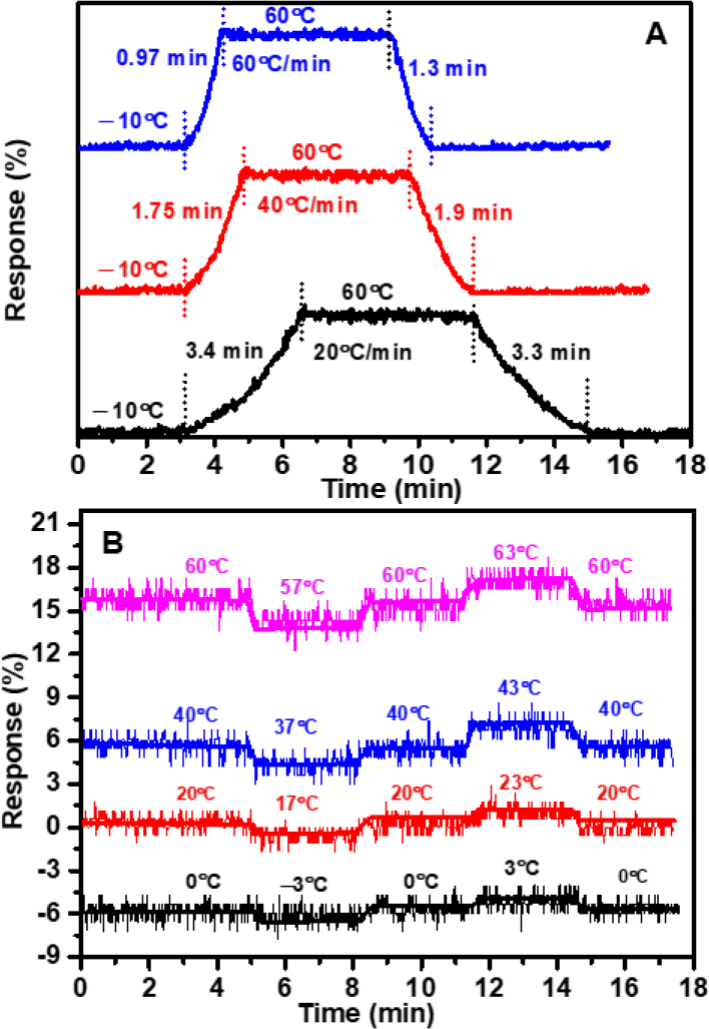
(A) Sensor response and recovery times under different heating/cooling rates of 20, 40, and 60 °C min^–1^. (B) Sensor sensitivity to ±3 °C fluctuations at 0, 20, 40, and 60 °C temperatures.

However, it is notable that the sensor exhibited a significant baseline drift when operated at approximately 80% RH, particularly during the heating process. As the temperature increased from 0 to 60 °C, the sensor continued to exhibit a drift in response, reaching a response of 17.9% at 60 °C, approximately 2% higher than the sensor’s response of 15.9% in the dry environment (RH ≈ 10%). In contrast, a step-like response pattern was demonstrated when assessing the sensor’s response during the cooling process in the wet environment, similar to that observed in the dry environment, as shown in Figure 3A. Further analysis revealed that the sensor exhibited a distinct linear response region from 10 to 50 °C with a TCR ≈ 0.36% °C^–1^ (Figure 5C). This finding is consistent with our previous observations of the sensor’s response to varying temperatures in uncontrolled humidity conditions.^44^ The sensor responded at −3.6% at 10 °C and reached a response of 10.5% at 50 °C. Validating the sensor’s response behavior outside this range was challenging due to the risk of condensation and water molecules’ evaporation (condensation removal) at the sensor’s surface, leading to drift, as observed at 0 and 60 °C. These processes can disrupt the flow of electricity within the sensor, affecting its response behavior.

Consequently, this suggests that the sensor may be suitable for applications requiring temperature measurements between 10 and 50 °C, regardless of humidity levels, without needing modifications. One possible solution is to protect the sensor’s surface with a passivation layer, such as the fluorinated polymer passivation (CYTOP), to mitigate the baseline drift observed at RH ≈ 80% and low temperatures.^50^ Applying a passivation layer can enhance the sensor’s stability in environmental humidity by reducing the interference of humidity with the sensor’s performance.

This passivation layer can improve the sensor’s stability in humid environments by reducing the impact of humidity on its performance. This layer acts as a barrier between the sensor and its surroundings, minimizing the adsorption of water molecules on the sensor’s surface. Therefore, the passivation layer has the potential to enhance the accuracy and reliability of the sensor’s temperature measurements in humid environments, making it suitable for a range of applications.

To confirm our findings, we conducted dynamic response curve measurements for the temperature sensor in an environment with uncontrolled RH that fluctuated between 40 and 90% (Figure S5). Figure 3D depicts the resistance curve during the heating and cooling across a temperature range from −10 to 60 °C. Notably, the sensor’s dynamic response and recovery curves reveal an absence of significant baseline drift, even at varying temperatures. Figure 3E shows a clear linear relationship between the sensor’s resistance and temperature, with a TCR value of +0.3% °C^–1^. This TCR value represents the average for the three linear sensor sensitivities observed in dry and wet environments (i.e., Avg = ((0.12 + 0.42 + 0.36)/3) = 0.3% °C^–1^), as seen in Figure 3B,C,E.

The sensor’s response to temperature changes is intricately linked to its internal structure and the properties of its materials. The thermoplastic PS matrix serves as a structural component that significantly influences the sensor’s overall temperature responsiveness (Figure 4A). Embedded within this matrix, Gt flakes act as crucial conductive bridges (Figure 4A). The PS matrix maintains the film’s structural integrity and ensures the proximity of Gt flakes, facilitating efficient charge transfer within the sensor. This complex interplay between the structural dynamics of the material—particularly the role of Gt flakes as conductive bridges—and the influence of the thermoplastic PS matrix on the sensor’s electrical properties provides a reliable means of temperature measurement.

As the temperature increases, the PS matrix and the Gt flakes experience expansion (Figure 4B). This expansion separates the Gt flakes, reducing the probability of electrical charge transfer throughout the film. Consequently, this leads to a corresponding increase in the electrical resistance (increase in the sensor’s response). Conversely, the sensor’s resistance decreases as temperatures decrease (Figure 4B). This effect is due to the narrowing of the “grain boundaries” between Gt flakes within the film, which enhances electrical connectivity and reduces the sensor’s response.

Figure 5A provides a detailed view of the sensor’s response and recovery times when exposed to different heating and cooling rates, specifically 20, 40, and 60 °C min^–1^ at RH ≈ 10%. Notably, at a heating rate of 20 °C min^–1^, the Nextron testing chamber demonstrated excellent performance, taking only 3.5 min to transition from −10 to 60 °C. Impressively, our sensor’s response time of 3.4 min (3 s °C^–1^) and recovery time of 3.3 min (2.8 s °C^–1^) closely matched those observed in the Nextron test chamber under similar heating and cooling rates.

We conducted tests at higher heating and cooling rates of 40 and 60 °C min^–1^ to further evaluate the sensor’s performance. At a rate of 40 °C min^–1^, the Nextron chamber required 1.75 min to traverse the −10 to 60 °C range, during which our sensor exhibited response and recovery times of 1.75 and 1.9 min, respectively. Similarly, at a faster rate of 60 °C min^–1^, the chamber completed the same temperature range in 1.17 min, and our sensor demonstrated response and recovery times of 0.97 and 1.3 min, respectively. These results highlight the sensor’s ability to closely track and mirror the dynamic conditions of the environmental chamber (Figure 5A). Noteworthy, the heating/cooling rate of 60 °C min^–1^ represents the maximum capability of our environmental chamber. As a result, we were unable to measure the response time of our sensor beyond this limit.

Moreover, the sensor demonstrated notable sensitivity to ±3 °C fluctuations, a characteristic thoroughly examined at various temperatures, including 0, 20, 40, and 60 °C, as depicted in Figure 5B. The sensor’s response and recovery times highlight its ability to quickly and accurately adjust to temperature changes and return to a stable, reliable state. The sensitivity analysis further underscores the sensor’s precision and responsiveness in detecting temperature changes within a narrow tolerance range.

**Figure 6:**
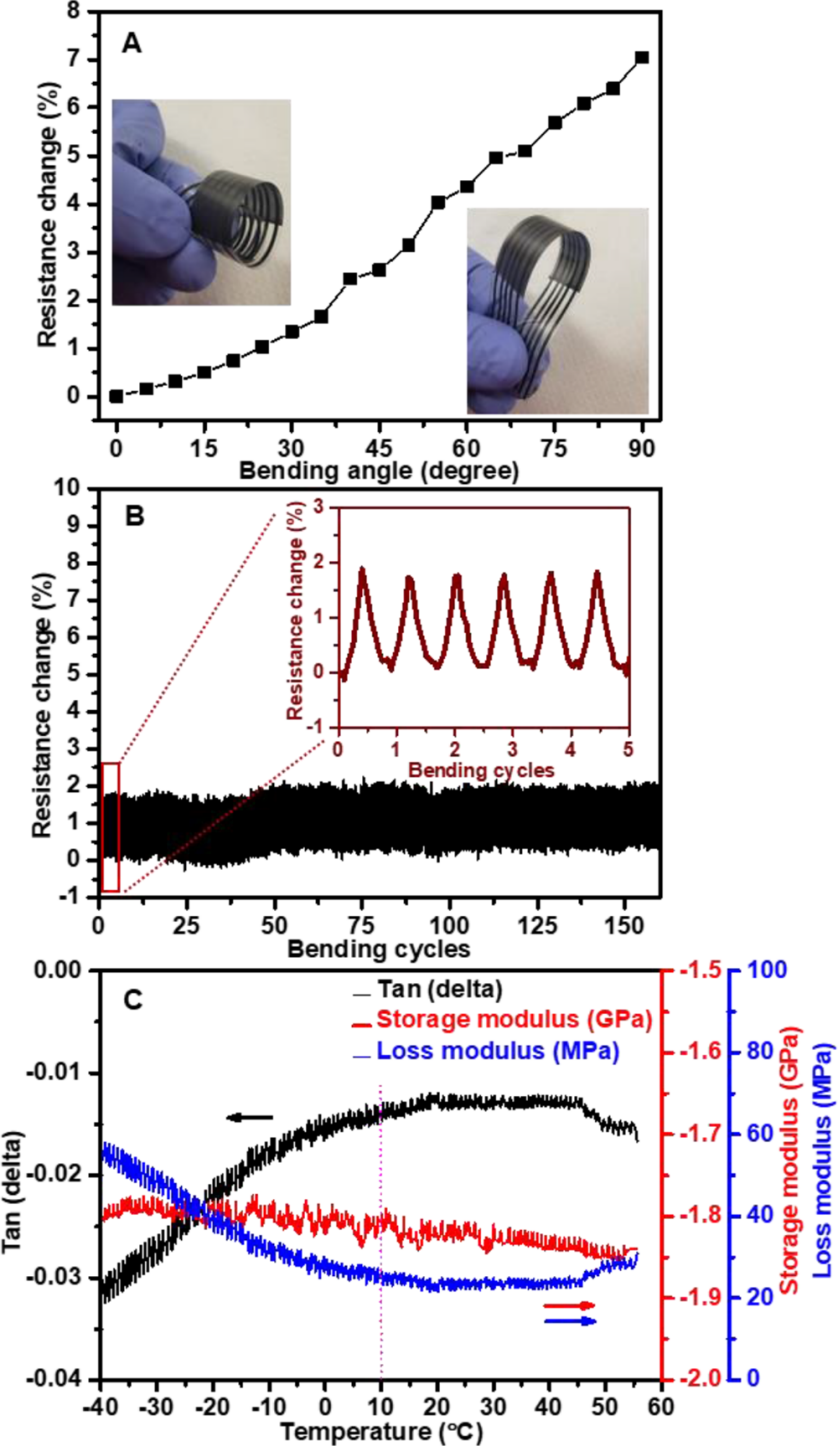
(A) Changes of the normalized resistance at different bending angles and (insets) for the temperature sensor. (B) Normal resistance changes during the repetitive bending test at bending angles of 30°. (C) DMA results showing tan(δ), storage, and loss modulus.

Investigating the sensor’s mechanical properties is important, particularly for wearable sensor applications. These sensors should exhibit key characteristics such as flexibility and thermomechanical stability to be effective in various IoT temperature-sensing applications. The sensor’s mechanical flexibility was initially assessed by identifying the critical bending angle under ambient conditions (RT and RH ≈ 30–40%) at which the sensor would experience electrical failure. The sensor’s electrical resistance gradually increased by up to 7% at a bending angle of 90°, as illustrated in Figure 6A. Our previous work confirmed the excellent mechanical flexibility of the bare carbon electrodes, with minimal resistance changes observed under similar conditions.^51,52^

This finding indicates that the observed change in electrical resistance was primarily due to the distortion and twisting of the sensing layer rather than the carbon electrode itself. The sensor’s flexibility was further evaluated by subjecting it to repeated bending cycles at a chosen angle of 30° (bending radius ∼1 cm). The sensor displayed adequate performance, with a minor resistance change (1.5%) even after undergoing hundreds of bending cycles at this angle, as depicted in Figure 6B.

The bending angle of 30° can be sufficient to maintain the sensor’s structural integrity and sensitivity for temperature monitoring in applications that typically require moderate bending, where the sensor needs to conform to curved surfaces without significant performance degradation. As will be discussed later, improvements can be made for applications requiring greater flexibility or higher bending angles.

We evaluated Tan(d) in dynamic mechanical analysis to estimate the sensor material’s response to mechanical stress and temperature fluctuations. Figure 6C illustrates a minimal tan(δ) increase from −0.035 to −0.015 as the temperature rises from −40 to 10 °C, followed by a phase of stability up to 60 °C. The storage modulus displayed a great consistency over the entire temperature span from −40 to 60 °C. This behavior signifies that the material’s stiffness and elasticity remained relatively unaltered within this thermal range. Alternately, the transition in the loss modulus, decreasing from 60 to 20 MPa as the temperature increased from −40 to 10 °C, followed by stabilization up to 60 °C, represents a shift in the material’s viscoelastic behavior in response to temperature variations. The subsequent stabilization of the loss modulus with rising temperatures indicates that, within this specific temperature range, the material maintains a relatively consistent capacity for energy dissipation.

Overall, the DMA analysis results confirm that the material is resilient to temperature variations within the tested limits and does not undergo significant phase transitions or internal structural changes. The stability of the thermomechanical properties (loss/storage moduli and tan(δ)) makes this sensor a valuable candidate for applications where consistent mechanical behavior across a wide range of temperatures is essential.

**Figure 7:**
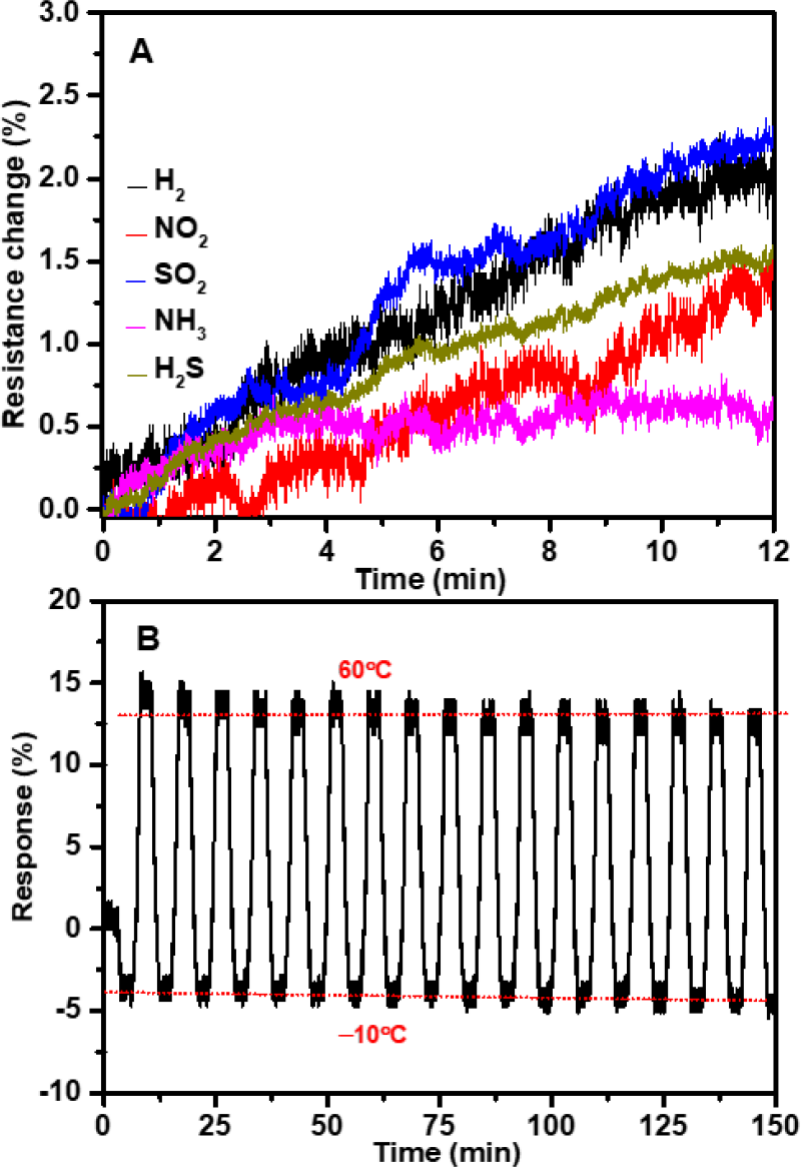
Evaluation of the sensing thin film’s chemical and physical stabilities. (A) Response of the sensor’s electrical resistance to corrosive gases at 4 ppm concentration. (B) Sensor sensing performance was measured between −10 and 60 °C at RH 10% after a month of immersion in tap water and (inset) a photo of the immersed sensor.

Moreover, we investigated the sensor’s chemical and physical stabilities through two distinct approaches. First, the sensor was exposed to corrosive gases (NO_2_, SO_2_, H_2_, H_2_S, NH_3_) at a concentration of 4 ppm under ambient conditions for each gas, with each exposure lasting 12 min. We closely monitored the sensor’s electrical resistance throughout this process, as illustrated in Figure 7A. Remarkably, the sensor exhibited only a 2% change in resistance, indicating excellent chemical stability against these corrosive gases, even without an encapsulation layer.

In a separate evaluation, we submerged sensors entirely in tap water for several weeks, as depicted in Figure 7B. Each week, we retrieved one sensor for SEM imaging and subjected it to temperature testing within the range of −10 to 60 °C at RH 10%, comparing its performance to that of a fresh sensor. Intriguingly, despite the gradual accumulation of minerals on the sensor’s surface, as detected during weekly SEM examinations (Figure S6A,C–G), the sensor consistently mirrored the performance of the fresh sensors. Additionally, as depicted in Figure S6F, EDS analysis revealed the presence of calcium, sodium, and magnesium salts on the sensor’s surface. This comprehensive investigation underscores the sensor’s robust chemical and physical stabilities, ensuring its reliability even in potential environments that simulate poor storage conditions.

The insights gained from these evaluations are crucial in ensuring reliable temperature measurements in practical applications. These experiments provided a vital testbed for assessing the sensor’s ability to accurately measure temperature changes, highlighting the importance of even minor temperature variations in various scenarios.

By simulating real-world conditions with common environmental fluctuations, we derived crucial findings that underscore the reliability and efficiency of the temperature sensor in practical applications.

**Figure 8:**
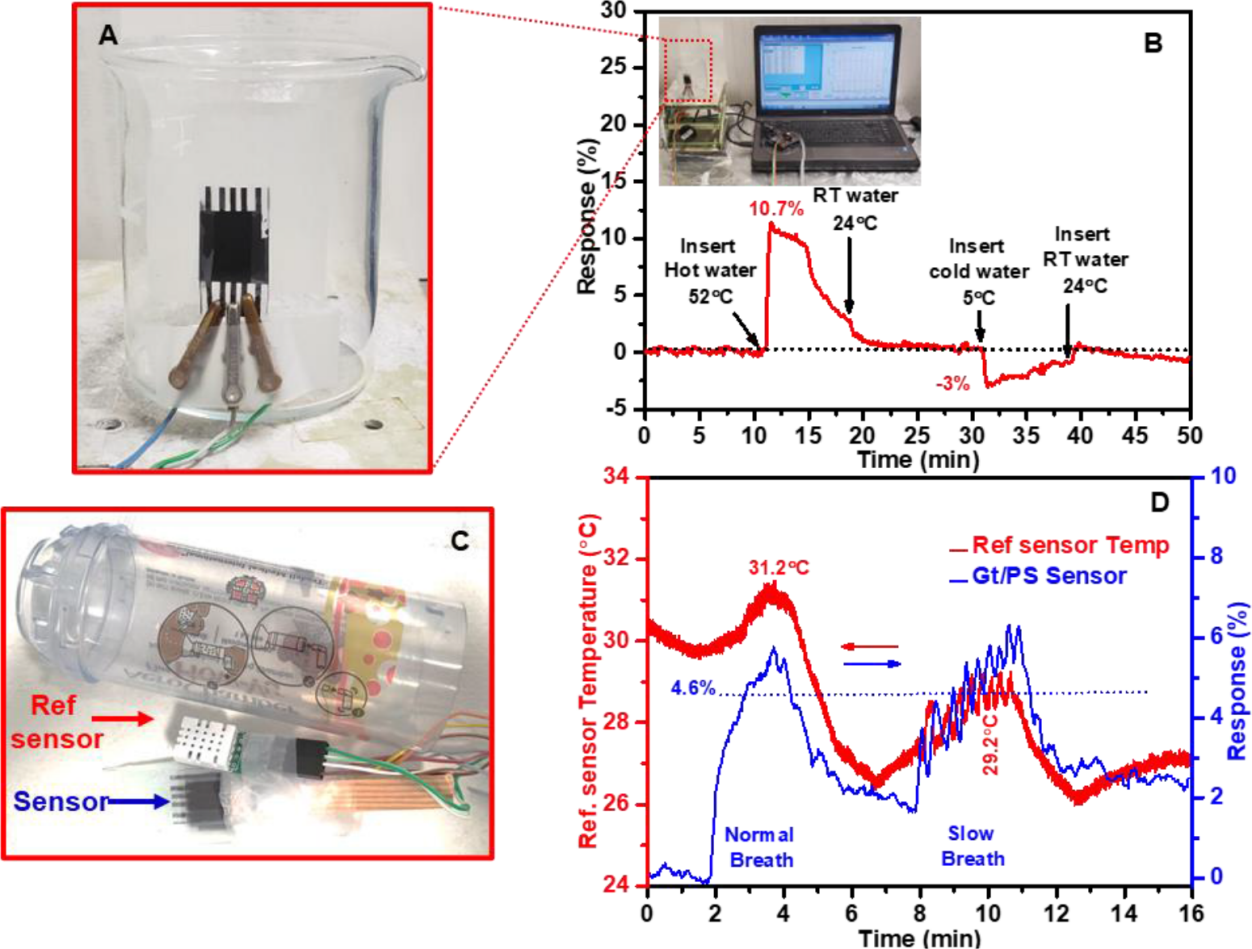
Real-world evaluation of temperature sensor performance. (A) Sensor installation on the beaker’s surface for measuring its response to varying water temperatures. (B) Sensor’s response to hot and cold water. (C) Sensor integration into an inhaler for breath temperature monitoring. (D) Sensor’s response to breath temperature.

In one experiment, the sensor was securely attached to the outer surface of a glass beaker as a solid object, seamlessly integrated with the measurement system. We then introduced water at different temperatures into the beaker, including hot water at ∼52 °C, RT water at ∼24 °C, and cold water at ∼3 °C, while closely sensor’s response (Figure 8A). After introducing hot water, the sensor exhibited a response of approximately 10.7% (Figure 8B), closely matching the temperature change and aligned well with the sensor response in an uncontrolled RH environment to temperature 50 °C ± 3 °C (Figure 3E). The sensor accurately tracked this transition when RT water was added to return to the initial temperature conditions. Upon introducing cold water, the sensor displayed a corresponding reduction in response, approximately −3% (Figure 8B), aligning once again with the temperature change of 6 °C ± 3 °C in an uncontrolled RH environment (Figure 3E).

In another approach, the sensor was used to monitor breath temperature. It was integrated into an inhaler spacer alongside a commercial Temp/RH reference sensor (DollaTek SHTC3), as depicted in Figure 8C. Unlike the beaker experiment, where the open environment minimized significant RH fluctuations around the sensor, the inhaler test presented a more challenging scenario. The confined space within the inhaler significantly increased the RH, as confirmed by the reference sensor’s readings (Figure S7). The PS-based temperature sensor demonstrated a ∼ 4.6% response to breath temperatures ranging from approximately 29.2 to 31.2 °C, as measured by the DollaTek reference sensor (Figure 8D). This response (∼4.6%) aligns well with the temperature change of 31 °C ± 3 °C in an uncontrolled RH environment (Figure 3E). Additionally, the sensor matched the performance of the reference temperature sensor in monitoring breath temperature during both normal and slow breathing patterns (with a 30-s interval between consecutive breaths). This highlights the sensor’s excellent temperature resolution and efficiency for healthcare applications.

Based on our analysis and discussions, the Gt/PS nanocomposite-based temperature sensor demonstrates competitive sensitivity compared to traditional Resistance Temperature Detectors (RTDs), which operate by measuring the change in electrical resistance of a metal with temperature. Examples of RTDs include Platinum RTDs, such as Pt100 and Pt1000, which are widely used due to their high accuracy and broad operational range (−200 to +850 °C), as well as Copper and Nickel RTDs, each suited for specific temperature ranges. Unlike RTDs, which often require complex construction methods such as thin-film, wire-wound, or coiled-element designs, our sensor employs a simple, cost-effective preparation process using the doctor blade technique. This straightforward fabrication method avoids the lengthy and costly procedures associated with RTDs, enabling scalability and practical implementation. Additionally, the Gt/PS sensor’s flexibility and stability make it more suitable for dynamic applications such as wearable electronics, where RTDs—limited by rigid structures or sensitivity to mechanical strain—may be less effective. Combined with the sustainability of its materials (Gt and recyclable PS), these features position the Gt/PS sensor for dynamic applications. Particularly in wearable electronics, IoT systems, and flexible devices, where RTDs may struggle due to rigid structures or sensitivity to mechanical strain. The Gt/PS sensor emerges as a versatile and low-cost alternative. These attributes, coupled with ongoing efforts to enhance response time and accuracy further, position the sensor as a desirable solution for modern temperature-sensing applications.

On the other hand, compared to previously published works, our temperature sensor presented in this manuscript offers several notable advancements compared to existing literature. Unlike typical sensors that use polymer,^1,53^ graphene,^21,22^ CNTs,^26^ or MOs,^27^ they are mainly limited to operating temperatures above room temperature and are sensitive to environmental conditions like gases and humidity. Our sensor works effectively in a broader range from subzero (−10) to 60 °C and demonstrated a clear chemical, physical, mechanical, and thermomechanical quality to maintain temperature monitoring efficiency in real work applications. Moreover, the sensor’s fabrication method, utilizing doctor blade coating, is suitable for large-scale printing, offering a more cost-effective and scalable solution than the more complex techniques commonly used in sensor production.

Table 1 focuses on the performance parameters of flexible temperature sensors tested in the subzero range. Our Gt/PS-based temperature sensor demonstrates a balanced performance across key parameters. Operating in the −10 to 60 °C range, it achieves a sensitivity of 0.3% °C^–1^, which is comparable to sensor based on Ag NWs (0.33% °C^–1^) and Ni (0.44% °C^–1^), but lower than that of carbon-based sensors, such as PDMS/graphene (4.87% °C^–1^) and AgNPs/CNT (2.99% °C^–1^).

**Table 1:** Performance Parameters of Flexible Temperature Sensors Tested in the Sub-Zero Temperature Range

materials	temperature range (°C)	sensitivity (% °C^–1^)	response/recovery	accuracy (±°C)	ref
Gt/PS	–10 to 60	0.3	0.97/1.3 min	3	This work
carbonized PI	–10 to 60	0.14			(54)
AgNPs/CNT	–20 to 40	2.99		0.1	(55)
PDMS/graphene	–40 to 300	4.87		0.5	(56)
Ag NWs	–20 to 20	0.33			(57)
Ni	–60 to 80	0.44	10 s		(58)
Mn–Co–Ni–O	–5 to 85	–3.9			(59)

However, while the current sensor demonstrates excellent repeatability, mechanical flexibility, and chemical stability, its sensitivity and ±3 °C resolution suggest it is suitable for broader temperature monitoring applications, where absolute precision is less critical and/or enhances its applicability in dynamic and challenging environments where traditional, more rigid sensors might not be practical. To address these issues (sensitivity and resolution), further refinements are planned to enhance accuracy and sensitivity. These include transitioning to higher *T*_g_ substrates (PI substrates) or nanocomposite formulations explicitly tailored for such applications, adapting and applying strategies to our temperature sensor design, incorporating calibration protocols specific to the operating environment, integrating advanced signal processing techniques to reduce noise, and applying protective coatings to mitigate interference from environmental factors like humidity.

### Conclusions

This study developed a Gt/PS nanocomposite-based temperature sensor with characteristics suitable for various practical applications. The straightforward preparation method, producing an ink well-suited for sensor coating. The sensor demonstrated thermal stability under optimized curing conditions of 180 °C and 15 min, consistent repeatability, and a reversible response within a temperature range of −10 to 60 °C. It exhibited positive TCR values of 0.12 and 0.42% °C^–1^ within the operational temperature range at 10% RH and a TCR of +0.36% °C^–^1^^ at 80% RH. Moreover, the sensor maintained a TCR of +0.3% °C^–1^ in an uncontrolled RH environment, consistent with its performance under dry and wet conditions. The response and recovery times closely matched the heating and cooling rates of the testing chamber. The sensor also showed sensitivity to ±3 °C temperature fluctuations, making it suitable for applications requiring temperature monitoring. The Gt/PS nanocomposite sensor also exhibited mechanical flexibility, tolerating repeated bending cycles at a 30° angle without significant resistance changes. DMA results confirmed the sensor’s stability across a temperature range of −40 to 60 °C, indicating its robustness for potential use in dynamic environments. The sensor displayed excellent resistance to various corrosive gases (NO_2_, SO_2_, H_2_S, NH_3_) and maintained its functionality even after prolonged immersion in tap water, showcasing its robust chemical and physical stability. These characteristics make the sensor well-suited for harsh environments. Real-world testing confirmed its ability to accurately track temperature changes in various scenarios, including monitoring solid objects and human breath, which aligns with its calibration in uncontrolled humidity. Further refinements, including using higher *T*_g_ PI substrates, tailoring nanocomposite formulations, applying calibration protocols, integrating advanced signal processing, and using protective coatings, are planned to enhance the accuracy and sensitivity of the sensor.

## Supporting Information Available

FTIR spectra of xylene evaporate versus time at RT and ambient humidity; decomposition behavior of PS and Gt and their nanocomposites cured at different temperatures; RH fluctuations in dynamic response experiment; SEM and EDS analysis of sensor surface after prolonged immersion in tap water; and RH and temperature fluctuations throughout the breathing analysis test (Nextron, DollaTek SHTC3, PS sensor) (PDF)

## Abbreviations

PS: polystyrene
Gt: graphite
PET: polyethylene terephthalate
SEM: scanning electron microscope
EDS: energy-dispersive X-ray spectroscopy
TGA: thermogravimetric analysis
FTIR: Fourier transform infrared spectroscopy
RH: relative humidity
TCR: temperature coefficient of resistance
DMA: dynamic mechanical analysis
HSPs: Hansen’s solubility parameters
Da: Dalton
mPa·s: milliPascal-second
mN m^–1^: milliNewton per meter
kΩ: kilohm
°C: degrees Celsius
min: minute
mL: milliliter
μm: micrometer
rpm: revolutions per minute
W: Watt
mm: millimeter
ppm: parts per million
mJ m^–2^: milliJoule per square meter
MP^1/2^: square root of megapascal
H_2_: hydrogen
NO_2_: nitrogen dioxide
SO_2_: sulfur dioxide
NH_3_: ammonia
H_2_S: hydrogen sulfide
*M*_w_: molecular weight
