# Supplementary material for: Fully Printed Flexible Polystyrene/Graphite-Based Temperature Sensor with Excellent Properties for Potential Smart Applications
Source: ACS Omega. 2025 Jan 23;10(4):3878–89. doi: 10.1021/acsomega.4c09548 (PMC11800034; doi:10.1021/acsomega.4c09548)
Supplement: Supplementary file 1 — ao4c09548_si_001.pdf [file ao4c09548_si_001.pdf]

Supporting information

Fully Printed Flexible Polystyrene/Graphite-Based Temperature Sensor with Excellent Properties for Potential Smart Applications

Ahmad Al Shboul,[1,*] Mohsen Ketabi,[1] Jenner H. L. Ngai,[2] Daniella Skaf,[3] Simon Rondeau-Gagné,[3] and Ricardo Izquierdo.[1,*]

[1] Department of Electrical Engineering, École de Technologie Supérieure (ETS), 1100 Notre-Dame St W, Montreal (QC), H3C 1K3, Canada

[2] Security and Disruptive Technologies (SDT) Research Centre, National Research Council of Canada, 1200 Montreal Road, Ottawa, Ontario K1A 0R6

[3] Department of Chemistry and Biochemistry, Advanced Materials Centre of Research, University of Windsor, Windsor, ON, Canada N9B 3P4.

Decomposition Behavior at Various Curing Temperatures

The thermal stability of the Gt/PS nanocomposite was systematically evaluated and optimized through TGA analysis in ambient air, focusing on the effects of curing temperature and duration. **Figure S1(A)** demonstrates that raw PS material exhibited a single-step decomposition pattern between 200°C and 355°C, while raw Gt powder decomposed between 550°C and 800°C. In contrast, pure PS films were prepared from a 250 mg/mL PS solution in xylene. They were cured at different temperatures (60°C to 210°C) for one hour and displayed a distinct three-step decomposition pattern (**Figure S1(B)**). This pattern varied with curing temperature, as lower curing temperatures (60°C - 120°C) resulted in higher weight loss (0%–9.5%) between 65°C and 220°C, attributed to the decomposition of low-molecular-weight PS. At higher curing temperatures (150°C - 210°C), no weight loss was observed between 65°C and 220°C due to the sublimation of low-molecular-weight PS during curing, enhancing thermal stability.

For the Gt/PS Nanocomposites (**Figure S1(C)**), the decomposition behavior also shifted significantly with curing temperature. At lower curing temperatures (60°C and 90°C), a three-step decomposition pattern was observed: initial weight loss (6.8%–9.5%) between 65°C and 220°C due to low-molecular-weight PS breakdown, followed by 73%–77% weight loss from PS decomposition between 220°C and 330°C, and final decomposition (17.5%) of Gt powder between 520°C and 700°C.

For the Gt/PS Nanocomposites (**Figure S1(C)**) cured at 120°C and 150°C displayed a two-step decomposition pattern, with the primary PS decomposition (82.5% weight loss) occurring between 220°C and 330°C, and Gt powder decomposition (17.5%) between 520°C and 700°C. Notably, curing at 150°C elevated the decomposition onset to 580°C, suggesting enhanced thermal stability due to Gt/PS complexation.

When cured at higher temperatures (180°C and 210°C), the nanocomposite showed significant thermal stability improvements, with decomposition shifting to elevated ranges: 270°C–430°C for PS (82.5% weight loss) and 630°C–810°C for Gt powder (17.5% weight loss). These findings indicate that optimal thermal stability is achieved at curing temperatures of 180°C and 210°C.

The curing duration at 180°C was also optimized (**Figure S1(D)**). Nanocomposites cured for 5 minutes exhibited a three-step decomposition pattern with weight losses of 3.7%, 78.8%, and 17.5% in the ranges of 110°C–270°C, 270°C–430°C, and 630°C–810°C, respectively. For longer curing times between 10 minutes and 1 hour, the pattern transitioned to a two-step decomposition, with 82.5% and 17.5% weight losses in the ranges of 270°C–430°C and 630°C–810°C, respectively. This optimization highlights the role of curing temperature and duration in tailoring the thermal stability of the Gt/PS nanocomposite for enhanced performance.

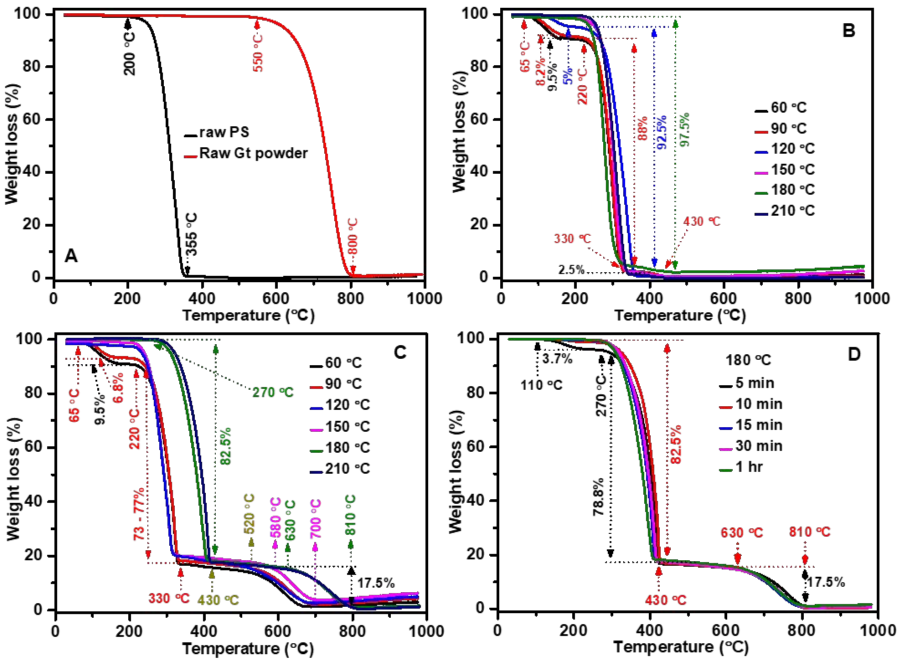

Figure 2. (A) Decomposition behavior for raw PS and Gt powder materials, and (B) pure PS films at different curing temperatures for one hour (60, 90, 120, 150, 180, and 210 °C). (C) Thermal analysis and decomposition behavior of Gt/PS nanocomposite cured at various temperatures (60, 90, 120, 150, 180, 210°C) for 1 hour, and (D) Thermal analysis and decomposition behavior of Gt/PS nanocomposite cured at 180°C for different durations.

The morphological analysis

The morphological analysis of the fabricated sensors, prepared at varying curing temperatures, was conducted using SEM. As depicted in **Figure S2**, the examination revealed that varying the curing temperatures had no noticeable effect on the smoothness of the sensors or the integrity of their thin films. Based on the TGA and SEM analysis, a curing temperature of 180℃, maintained for 15 minutes, was consistently used for the sensor's preparation as the optimal curing procedure. This choice balances the desired thermal stability and the thin film's smoothness and integrity.

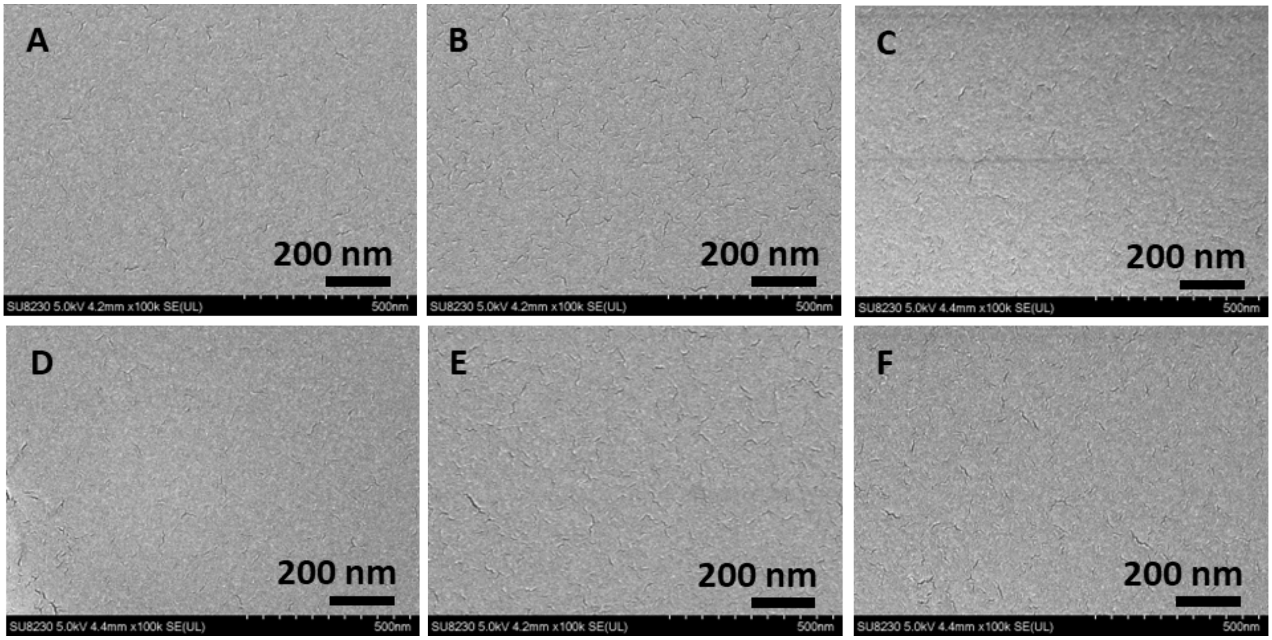

Figure S2. The morphological analysis of the fabricated sensors cured at various temperatures (A) 60℃, (B) 90℃, (C) 120℃, (D) 150℃, (E) 180℃, (F) 210℃.

Graphitization degree of graphite flakes (i.e. quality)

The spectral analysis presented in **Figure S3(A,B)** provides valuable insights into the structural and compositional properties of the sensor material compared to its precursors. The Raman spectra in **Figure S3A** display distinct peaks corresponding to the PET substrate, raw Gt, raw PS, and the final sensor material, underscoring the successful integration of components. The Gt spectrum exhibits the characteristic D-band and G-band peaks, with an intensity ratio ($I_D/I_G = 0.27$), indicating a relatively low defect density in the graphitic structure. The PS spectrum reveals its unique vibrational modes, confirming its role as the polymer backbone. The sensor spectrum combines Gt and PS features and additional peaks (marked with *), which likely signify interactions between the components during sensor preparation. These newly emerged peaks suggest successful chemical modification.

Furthermore, the Raman spectrum analysis in **Figure S3B** reaffirms preserving the graphitization degree of the graphite flakes, as evidenced by the consistent intensity ratio ($I_D/I_G = 0.27$). This observation highlights that the straightforward mixing process of graphite with polystyrene, without chemical modification, allowed the graphite flakes to retain their original sp^2 structure. These results demonstrate the sensor material's practical synthesis and structural evolution, which is critical for its intended application.

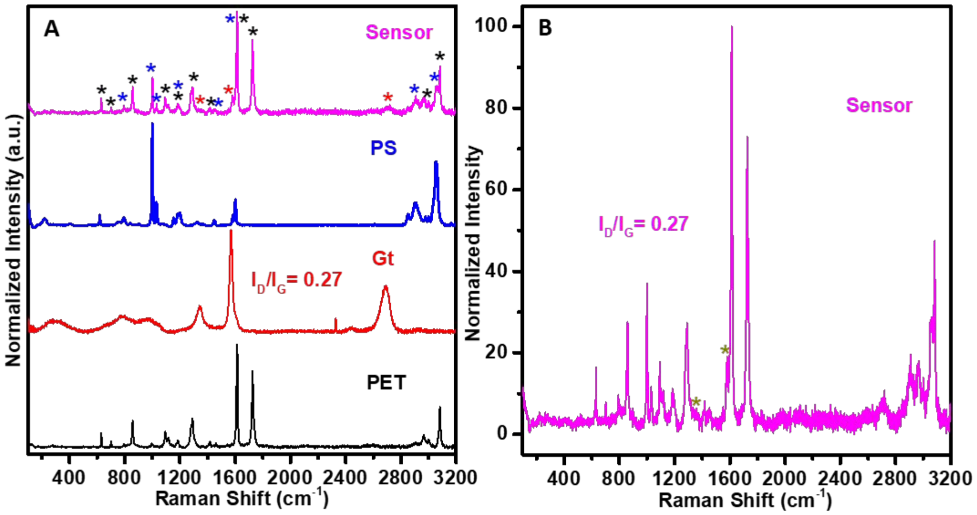

Figure S3. Raman spectroscopy of (A) PET substrate, raw Gt flakes, raw PS, and sensing thin film for a Gt/PS-based temperature sensor, and (B) high magnification of the Raman spectrum of the sensing thin film.

Drying rate measurement

To evaluate the drying rate of xylene **Figure S4(A,B)** , we deposited 50 µl of the pure solvent onto the ATR crystal (diamond) of the FTIR instrument and automatically saved the absorbance spectrum every 2 seconds. The drying rate was monitored by selecting a characteristic solvent band (specifically, the 1455 cm^{-1}), as shown in **Figure S4A**. Once this band disappeared, we determined that the xylene had thoroughly dried. Our results indicate that xylene had dried within a mere 2.7 minutes, as shown in **Figure S4B**.

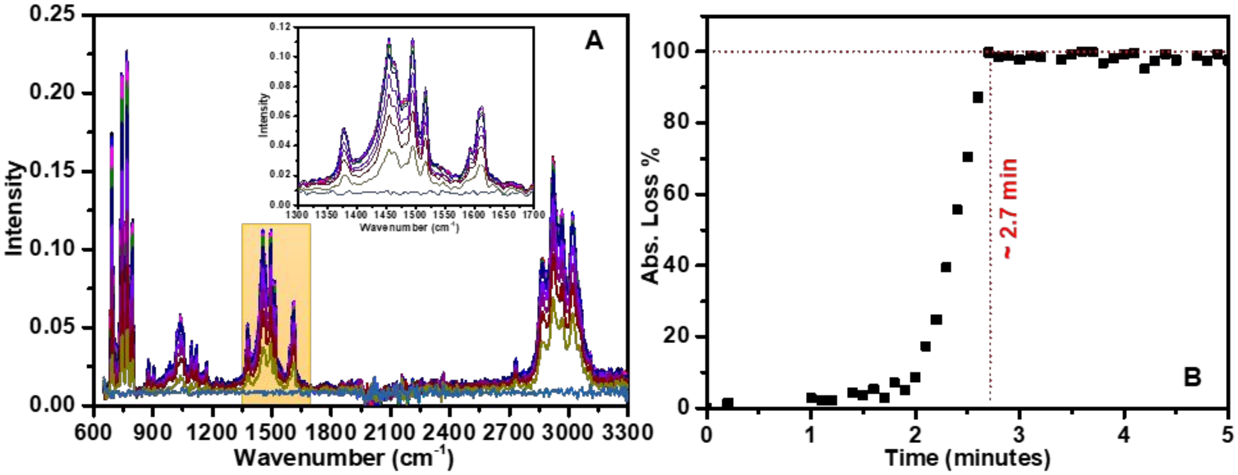

Figure S4. (A) Time-dependent FTIR spectra of xylene, with an inset showing the FTIR spectrum of xylene in the range of 1300 - 1700 cm^{-1}. (B) Percentage loss of xylene absorbance intensity over time.

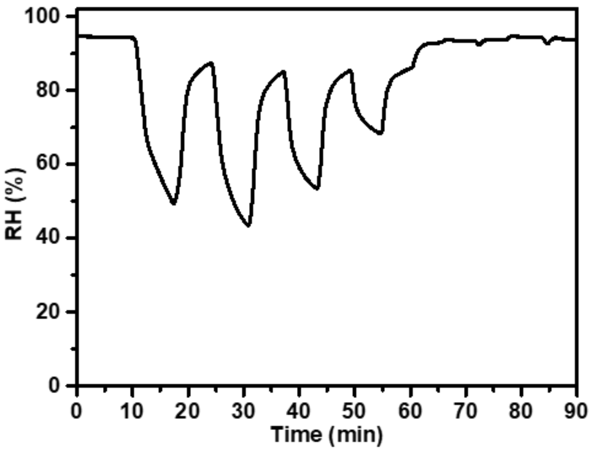

Figure S5. RH fluctuations in the dynamic response curve measurements as measured by the Nextron test chamber

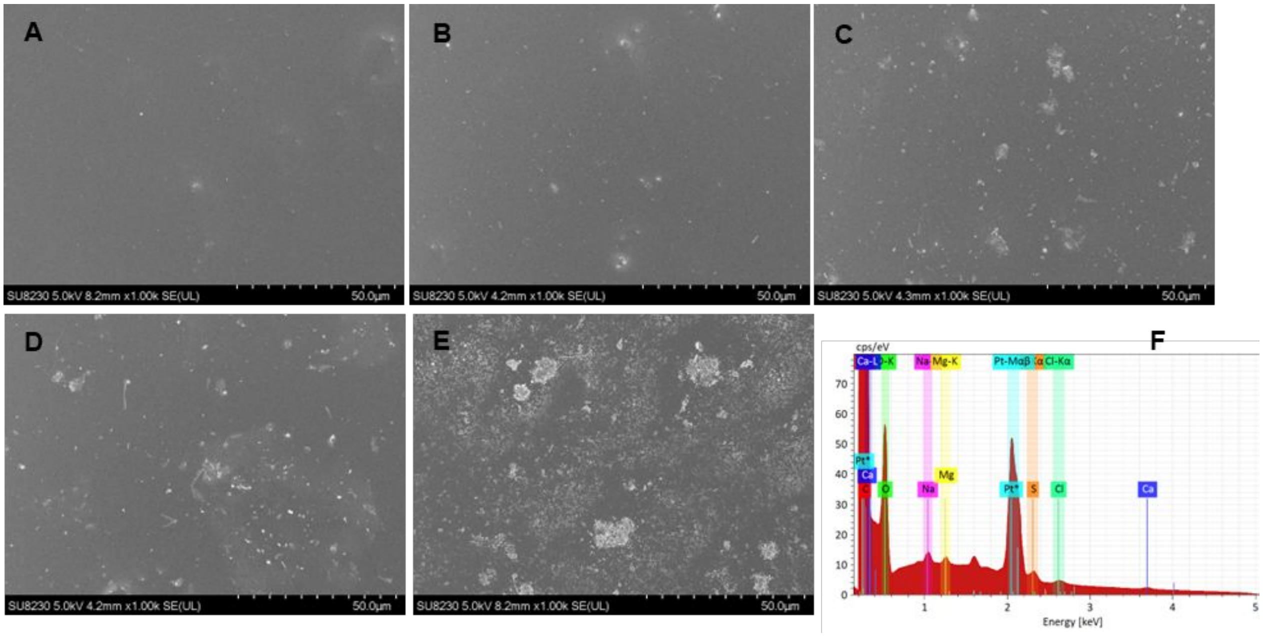

Figure S6. SEM images of the sensor's surface: (A) Fresh sensor, and after immersion for (B) 1 week, (C) 2 weeks, (D) 3 weeks, and (E) 4 weeks, depicting the gradual accumulation of minerals on the sensor's surface. (F) EDS analysis reveals the presence of calcium, sodium, and magnesium salts on the sensor's surface.

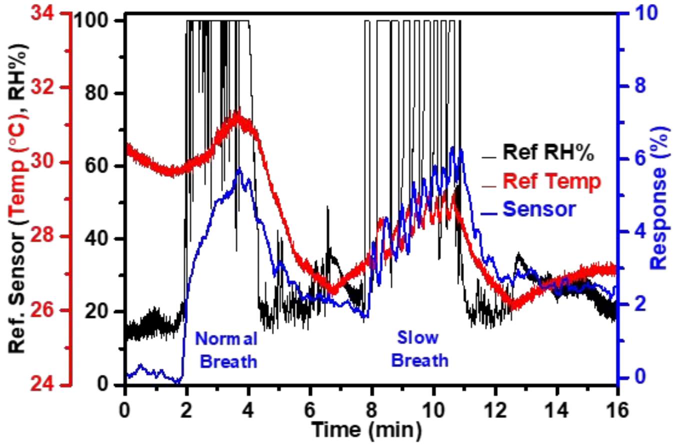

Figure S7. A commercial Temp/RH reference sensor (DollaTek SHTC3) and the PS-based Temperature sensor measure RH and temperature fluctuations in human breath.
